# A Comparison of Damages Occurring on the Bonding Surface of Carbon and Glass Fiber-Reinforced Polymer Composite Materials Used in Wind Turbine Blades and Marine Vessels via Three-Point Bending and Four-Point Bending Tests

**DOI:** 10.3390/polym18040481

**Published:** 2026-02-14

**Authors:** Dudu Mertgenç Yoldaş, Gürcan Atakök

**Affiliations:** 1 Department of Mechanical and Metal Technologies, Izmir Vacotional School, Dokuz Eylul University, Buca, 35360 Izmir, Turkey; 2 Department of Mechanical Engineering, Faculty of Technology, Marmara University, Maltepe, 34854 Istanbul, Turkey; gatakok@marmara.edu.tr

**Keywords:** adhesive bonding, composite materials, diffusion, four-point bending test, three-point bending test

## Abstract

The aim of this study is to experimentally evaluate the damage mechanisms occurring in the adhesive-bonded regions of glass fiber-reinforced polymer (GFRP) and carbon fiber-reinforced polymer (CFRP) composites, which are widely used in marine and offshore wind turbine applications, under environmental conditions. In particular, this study focuses on the degradation caused by long-term seawater exposure and its effects on the bending behavior and load-carrying capacity of adhesive joints. For this purpose, the specimens were prepared in accordance with ASTM D5868-01, using 7-layer GFRP and 8-layer CFRP laminates. Single-lap adhesive joints were fabricated. To simulate marine environmental conditions, the single-lap adhesive joints were immersed in natural seawater obtained from the Aegean Sea (22 °C temperature and 3.3–3.7% salinity) for 1, 2, and 3 months in separate containers. Three-point bending (3PB) tests were performed on specimens representing marine applications, while four-point bending (4PB) tests were conducted on specimens representing offshore wind turbine blade structures. The results quantitatively revealed the influence of seawater on adhesive-bonded composite joints. In 3PB tests, the reductions in the Young’s modulus of GFRP specimens after 1, 2, and 3 months of exposure were measured as 5.94%, 8.90%, and 12.98%, respectively. For CFRP specimens, degradation was more limited, with corresponding reductions of 1.28%, 3.39%, and 3.74%. A similar trend was observed in 4PB tests representing offshore wind turbine applications, where GFRP joints exhibited modulus reductions of 3.15%, 6.42%, and 9.45%, while CFRP joints showed reductions of 1.29%, 2.62%, and 3.48% for the same exposure durations. Overall, the findings demonstrate that CFRP composites exhibit more stable mechanical behavior under environmental exposure, whereas GFRP structures undergo more pronounced performance losses, particularly in moisture- and salt-rich environments. These results highlight the critical importance of material selection for long-term durability in offshore composite structures. The outcomes of this study contribute to a better understanding of the damage processes occurring in composite adhesive joints under environmental conditions and provide a scientific basis for developing more reliable design and material selection strategies in both the marine and wind energy sectors.

## 1. Introduction

In modern engineering applications, composite materials, which are synthetic, multi-phase systems produced by combining two or more different phases within a defined structure, are increasingly preferred. In this context, both synthetic and natural fiber-reinforced composites are utilized [1,2]. Composites can simultaneously fulfill multiple functions in modern applications by combining the lightness and flexibility of the matrix with the superior properties of the reinforcing fillers [3].

To meet specific requirements, the adjustment of electrical properties involves the selection of compression pressure, particle size, and composition. In these materials, there exists a clearly defined interface between phases, which provides enhanced performance characteristics that cannot be achieved by individual components alone. Due to their advantages such as lightness, high strength, flexibility, and environmental resistance, composites have gained strategic importance [4,5].

The effective and safe use of these materials is not limited to understanding the properties of their components but also requires an understanding of how these properties contribute to the overall material integrity. The behavior of composites becomes complex when subjected to variable external conditions such as impact, temperature fluctuations, vibration, and cyclic loading. Therefore, fiber-reinforced composites are widely preferred in aerospace and other engineering fields due to their excellent specific strength, stiffness, and corrosion resistance. At this point, the interfacial region that ensures load transfer within the material is of vital importance. The structural integrity of the interface enables efficient load transfer, while weaknesses in this region can negatively affect the overall durability of the structure [6,7].

Composites formed by combining phases with different physical and chemical properties in an orderly manner offer both structural and functional advantages in various engineering fields. These materials, which stand out for their high strength-to-weight ratio, formability, and resistance to environmental conditions, are widely used in aircraft and automotive industries, construction, energy systems, and defense technologies. In tribological applications, environmental conditions should also be considered when selecting GFRP and CFRP [4,8,9].

Fiber-reinforced polymer (FRP) composites are widely preferred for both wind turbine blades and marine structural components due to their light weight and high structural strength. While the production of large-span wind turbine blades from these materials increases energy efficiency, because the structures are often composed of multiple composite parts bonded with adhesives, the interface and bonding zones are at risk of failure over time under environmental influences and cyclic loading [10,11,12]. Similarly, in FRP structures used in marine environments, conditions such as saltwater, wave forces, UV radiation, and temperature changes can weaken the interface strength and pose critical challenges to long-term structural integrity [13,14,15]. Therefore, understanding the failure mechanisms of bonding surfaces and interface zones in both turbine blades and marine structures is crucial for ensuring long-term performance. The sustainability of structural integrity depends not only on the mechanical properties of the reinforcing fibers and matrix but also on the quality of their interaction, the homogeneity of the interface, and the accuracy of manufacturing techniques. Under dynamic loading, temperature variations, and wind effects, the behavior of the interface plays a decisive role. In particular, fiber orientation directly affects load distribution, shaping the overall performance of the structure [13].

Similarly, FRP structures used in marine environments are exposed to harsh conditions such as wave forces, the chemical effects of seawater, and thermal cycles; therefore, the strength of adhesive and interfacial regions constitutes a critical design issue in marine engineering as well [14,15]. Hence, comparing carbon fiber-reinforced composites with glass fiber-reinforced composites will enable a better understanding of their superior properties [16].

Today, fiber-reinforced polymer (FRP) composites are among the fundamental materials preferred in both wind turbine blades and marine structural components. In wind turbines, the lightweight and high-strength nature of large-span blades not only enhances energy efficiency but also challenges structural durability. Since these structures are mostly composed of multiple composite parts joined by adhesive bonding, interfacial and bonding regions are at risk of degradation over time under environmental effects and cyclic loading. Similarly, FRP structures used in marine environments face critical design challenges related to interface strength due to exposure to wave forces, saltwater, ultraviolet radiation, temperature changes, and repeated mechanical loads [13,17,18].

In this context, understanding the primary damage modes occurring in composite materials is a critical requirement for both performance evaluations and long-term durability analyses. FRP structures may develop various types of damage depending on loading conditions, environmental exposure, and manufacturing parameters. Below, a literature review of the primary damage modes of the composite materials analyzed in this study is presented. Delamination is characterized by the initiation and propagation of separation between fiber layers and is frequently observed under bending, impact, and fatigue loading conditions [19,20,21,22]. Manufacturing-induced voids, resin-rich or resin-deficient regions, and hygrothermal aging significantly accelerate the onset of delamination [20,21]. Fracture mechanics-based approaches and cohesive zone models are widely employed to model this type of damage [22,23]. The degradation of the fiber–matrix interface represents a critical damage mechanism that directly affects load transfer efficiency. Exposure to saltwater, UV radiation, and thermal cycling reduces interfacial strength and accelerates the formation of debonding [4,6,7]. Interfacial separation also facilitates the propagation of matrix cracks, which may evolve into larger-scale delamination [24,25,26,27]. Matrix cracking initiates at the microscale as a result of cyclic loading, environmental aging, and the accumulation of thermal stresses, and progressively leads to weakening of the fiber–matrix interface [21,25]. The coalescence of microcracks triggers the transition to more extensive damage modes such as delamination. These damage mechanisms include intralaminar cracking, cohesive damage, and shear-related failures [23,28]. Under realistic service conditions, damage in composite materials is rarely limited to a single mechanism; rather, delamination, matrix cracking, fiber breakage, and interfacial debonding often evolve in an interactive manner. Progressive damage analyses play a crucial role in understanding these multi-mode damage processes [21,23,26,28]. Wind turbine blades and marine structures are simultaneously exposed to multiple damage mechanisms due to environmental aging, hygrothermal effects, wave-induced loading, UV radiation, and repeated mechanical stresses. Therefore, an interactive assessment of delamination, interfacial debonding, and matrix damage is of critical importance for ensuring the structural reliability of such systems [25,26,27].

In addition to intralaminar and interlaminar damage mechanisms, recent studies have emphasized the significant influence of manufacturing techniques and joint configurations on damage initiation and propagation in composite structures.

Kadıoğlu (2025) focused on carbon fiber-reinforced thermoplastic single-lap joints and reported that co-cured configurations enhance damage tolerance and post-damage recovery through improved interfacial integrity and healing capability [14].

Sam-Daliri et al. (2025) investigated unidirectional glass fiber-reinforced epoxy composite joints manufactured using adhesive bonding and co-curing techniques, demonstrating that co-cured joints exhibit improved mechanical performance and more uniform stress transfer, thereby reducing damage susceptibility at the joint interfaces [29].

Similarly, Jiang et al. (2025) numerically analyzed adhesive-free glass fiber hydrofoil structures under flexural loading and showed that integrated, co-cured composite designs can sustain bending-induced damage without premature interfacial failure [29]. These studies collectively highlight that joint design and co-curing strategies play a critical role in controlling multi-mode damage evolution in composite structures, particularly under marine and energy-related service conditions.

In both application areas, microstructural interactions within the interfacial region, load transfer mechanisms, and manufacturing quality are critical factors in maintaining structural integrity. Accordingly, ensuring the long-term and reliable performance of composite structures used in wind turbine and marine environments requires comprehensive analyses and improvement strategies addressing the durability of both adhesive systems and interfacial materials [11]. A thorough understanding of damage formation and evolution mechanisms in interfacial regions under environmental exposure and cyclic mechanical loading is therefore of vital importance for the sustainability and safety of these technologies.

- **Applications of GFRP and CFRP Composite Materials in Offshore Wind Turbine Blades and Marine Vehicles**

**Figure 1:**
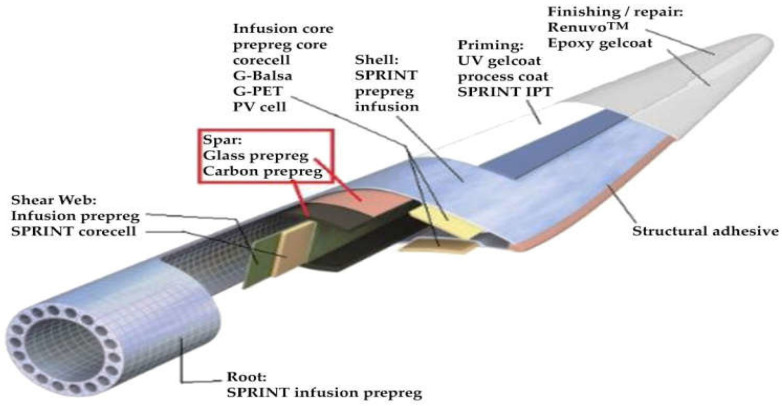
Material types according to structural components of wind turbine blade [34].

In wind energy technologies, blade design represents a critical engineering challenge in terms of both structural durability and energy efficiency. In particular, materials used in offshore wind turbine blades must withstand harsh environmental conditions such as high humidity, salinity, temperature fluctuations, and repeated mechanical loading over long service periods. In this context, glass fiber-reinforced polymer (GFRP) and carbon fiber-reinforced polymer (CFRP) composites are widely preferred in offshore wind turbines and marine structures due to their high specific strength, corrosion resistance, and manufacturing versatility [30,31,32,33]. GFRP composites are commonly used in the outer shell (skin) and surface layers of wind turbine blades owing to their low density, high impact resistance, and cost-effective production. In these regions, GFRP contributes to the overall structural stability of the blade by distributing aerodynamically induced stresses more uniformly. In contrast, CFRP reinforcements are typically employed in blade root and main spar regions, where higher bending stiffness and fatigue resistance are required. Due to their high elastic modulus and superior fatigue performance, CFRP materials reduce the overall weight of long blades and improve vibration characteristics [32,33]. Figure 1 illustrates the types of materials preferred in different structural regions of a wind turbine blade.

**Figure 2:**
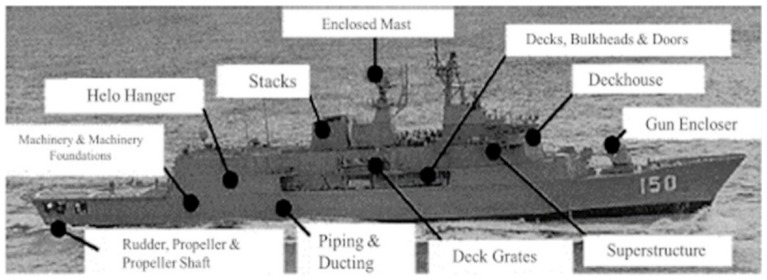
Applications of composite materials used in marine vessels [36].

In modern blade designs, hybrid lamination strategies combining GFRP and CFRP are increasingly adopted to achieve an optimal balance between mechanical performance and material cost. In such configurations, CFRP is used in high load-bearing layers, while GFRP is preferred for outer surfaces, thereby enhancing fatigue life while controlling material costs [35]. Similarly, the use of composite materials in marine applications has increased significantly due to advantages such as reduced structural weight, improved corrosion resistance, and lower maintenance requirements. GFRP has become a standard material for small- and medium-sized marine vessels, including yachts, boats, and submarine structures, owing to its ease of manufacturing, low cost, and high resistance to seawater. It is frequently employed in hull panels, deck structures, and internal structural components [14,19,20,21,22,23,24,25,26,27,28,29,30]. Figure 2 shows the main applications of composite materials in marine vessels.

CFRP, on the other hand, is mainly used in high-performance marine vessels, where its high specific modulus contributes to weight reduction, increased speed, and improved fuel efficiency. However, due to its susceptibility to galvanic corrosion when in contact with metallic fasteners, CFRP components require appropriate surface insulation layers or interfacial materials in such applications [32,33]. Seawater environments interact with FRP composites through both physical and chemical mechanisms, adversely affecting material stability and mechanical performance over time. Continuous moisture exposure, aggressive saline conditions, wave-induced loading, and repeated impact forces pose significant risks to the safety and service life of marine engineering structures [37]. Although polymer matrices provide partial protection to reinforcing fibers, moisture and ions can penetrate the composite through microcracks, voids, and fiber matrix interfaces. Moisture diffusion leads to matrix plasticization, swelling, and hydrolysis, resulting in degradation of mechanical properties [38]. Under hygrothermal conditions, these processes accelerate damage mechanisms such as anisotropic expansion, residual stress development, microcracking, delamination, and interfacial degradation [39]. Moisture diffusion behavior in FRP composites is commonly described using Fickian or anomalous (Langmuir-type) diffusion models, with Fickian diffusion generally considered dominant in thermoset-based systems. Recent experimental studies have demonstrated that moisture absorption significantly deteriorates not only the quasi-static properties of FRP composites, such as tensile, flexural, and interlaminar shear strength, but also their dynamic and viscoelastic responses [40]. These findings highlight the necessity of multiscale material optimization for marine and offshore wind energy applications. Accordingly, GFRP and CFRP composites used in offshore wind turbine blades and marine structures must be evaluated based on their region-specific loading conditions and environmental exposure. In marine structures, FRP components are often subjected to localized contact forces and irregular load transfer, which can be effectively represented by three-point bending (3PB) loading conditions. In contrast, loads in offshore wind turbine blades and bonded interface regions are distributed over a wider area, resulting in constant moment regions; therefore, four-point bending (4PB) loading regimes provide a more realistic representation of service conditions.

In this study, single-lap bonded specimens fabricated from seven-layer GFRP and eight-layer CFRP laminates were immersed under controlled conditions in natural seawater collected from the Aegean Sea, with a temperature of 22 °C and salinity levels ranging from 3.3% to 3.7%. Three-point bending tests were conducted on specimens representing marine applications, while four-point bending tests were applied to specimens representing offshore wind turbine interface regions. Following mechanical testing, microstructural damage mechanisms and deformation features in the bonded interface regions were examined in detail using scanning electron microscopy (SEM). The comparative results reveal the time-dependent effects of seawater exposure on damage initiation and evolution at the adhesive interfaces of GFRP and CFRP composites, providing reliable design data for both marine and offshore wind energy applications.

## 2. Materials and Methods

In this study, to obtain 2 mm thick laminate layers, the number of layers in GFRP and CFRP composites was determined by considering fiber-to-resin volume ratios and material densities. In this context, GFRP laminates with a 0/90° fiber orientation and a twill weave structure were composed of seven layers of 390 g/m^2^ glass fiber, while CFRP laminates were composed of eight layers of 245 g/m^2^ 3K carbon fiber.

Both materials used the same epoxy-based resin system (F-RES 21 resin and F-Hard 22 hardener mixed at a 100:21 ratio) and were produced using hand lay-up and hot pressing methods at 120 °C and 8–10 bar pressure for 60 min.

**Figure 3:**
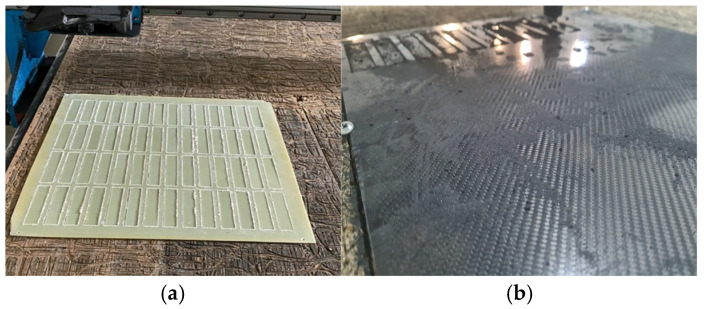
Machining of GFRP (**a**) and CFRP (**b**) Samples by CNC Router.

Prepreg production was performed with a drum-type machine, and the fiber-reinforced sheets were manufactured by Fibermak Engineering Company, located in İzmir, Turkey. The composite laminates were cut into 500 mm × 500 mm sheets using CNC machining, and the final laminate thickness was set to 2 mm (Figure 3). The mechanical properties of the laminates were determined as follows: tensile strength 80 MPa, tensile modulus 3300 MPa, flexural strength 125 MPa, and flexural modulus 3200 MPa.

**Figure 4:**
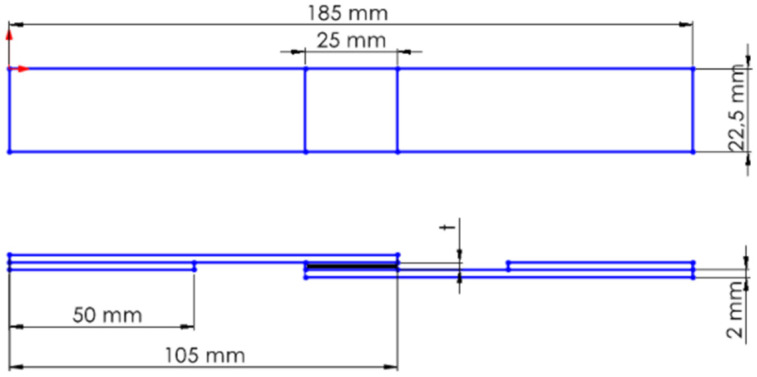
Schematic representation of GFRP and CFRP single-lap configurations.

**Figure 5:**
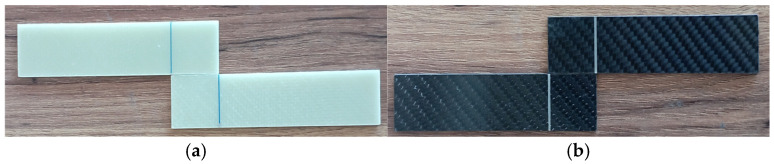
Marking of GFRP (**a**) and CFRP (**b**) single-lap configurations.

GFRP and CFRP specimens were then cut to the required dimensions in accordance with the ASTM D5868–01 standard [41] (Figure 4). Afterwards, 25 mm from the ends of the specimens were measured and marked (Figure 5).

**Figure 6:**
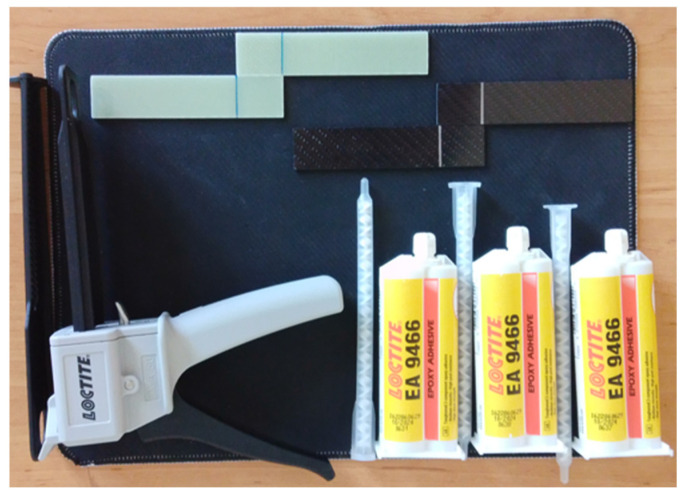
Bonding Process of GFRP and CFRP Specimens Using Loctite Hysol-9466.

The samples were prepared by bonding solvent-cleaned surfaces with adhesive. Bonding was achieved with two-component Loctite Hysol-9466 (Alpanhidrolik, Eskişehir, Turkey) epoxy, cured at room temperature and mixed at a 2:1 ratio in the applicator gun (Figure 6). The literature reports that adhesive layers of 0.1–0.3 mm thick provide high bond strength, while thicknesses greater than 0.6 mm reduce strength [42,43].

This is attributed to the fact that thin layers provide more effective mechanical resistance. After bonding, the samples were cured at room temperature for 7 days, in accordance with the product data sheet, and then moved on to the testing phase. This is attributed to the thin adhesive layers’ ability to support mechanical loads more effectively. Application was carried out under a constant pressure of 0.1 MPa and a target thickness of 0.2 mm; the evenness of the adhesive layer was confirmed by measuring it with a digital caliper.

**Figure 7:**
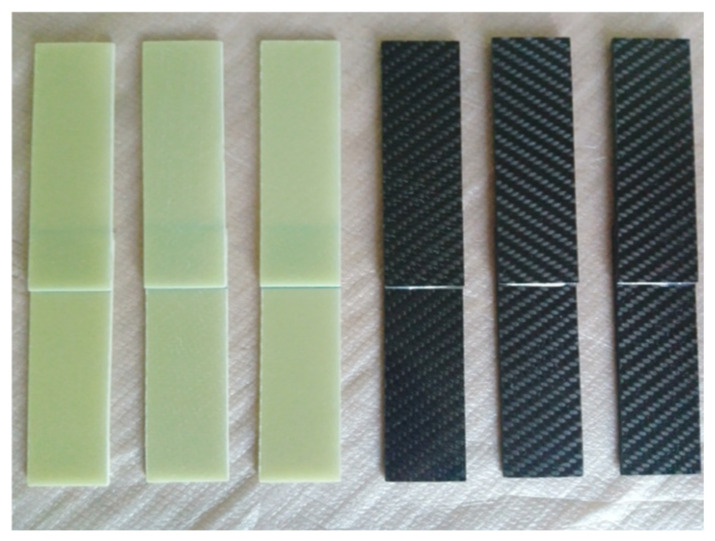
Visual observation of the surface morphology of GFRP and CFRP specimens prior to the three-point bending test.

**Figure 8:**
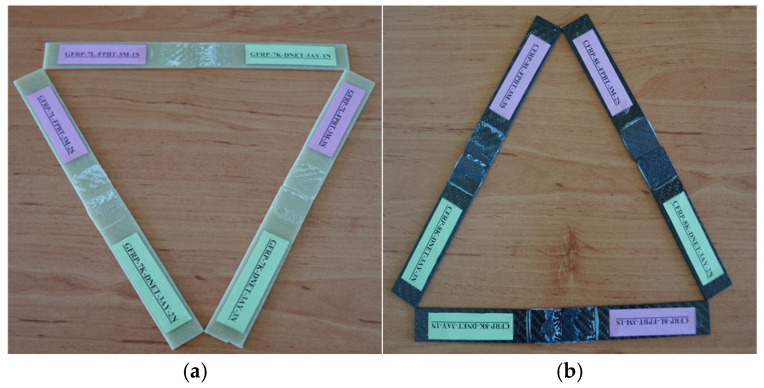
Visual observation of the surface morphology of GFRP (**a**) and CFRP (**b**) specimens prior to the four-point bending test.

General views of the surface areas of the GFRP and CFRP samples bonded with Loctite Hysol-9466 epoxy adhesive before the three-point and four-point bending tests are presented in Figure 7 and Figure 8, respectively. At this stage, it is observed that the adhesive layer is evenly distributed and a homogeneous bond is achieved between the samples.

In order to ensure that the experiments were conducted systematically and without confusion, distinctive codes were assigned to each specimen. The coding system used for the specimens tested in the three-point and four-point bending tests was organized to include the material type, number of layers, environmental conditions, and specimen sequence number. The following table presents example codes and explains their meanings (Table 1).

**Figure 9:**
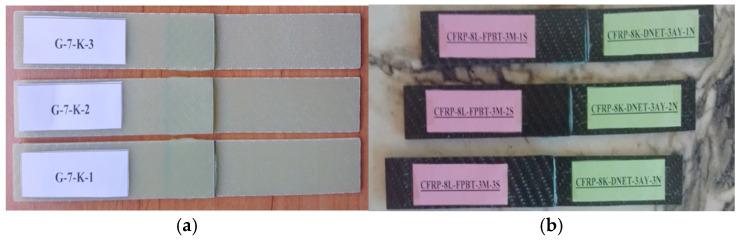
GFRP (**a**) and CFRP (**b**) specimen configurations used during three-point bending test.

For instance, the code G-7-K-1 refers to the first specimen made of glass fiber, consisting of seven layers, and tested in a dry environment (not exposed to seawater). Similarly, G-7-1A-1 represents the first specimen with the same properties but exposed to seawater for one month before testing. For the specimens made of carbon fiber, C-8-K-1 denotes the first eight-layer specimen tested under dry conditions (Figure 9).

**Figure 10:**
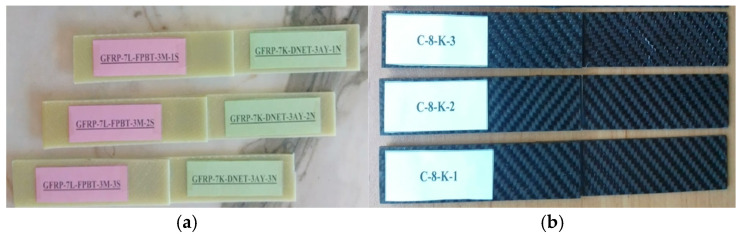
GFRP (**a**) and CFRP (**b**) specimen configurations used during four-point bending test.

In the specimens subjected to the four-point bending test, the coding system was arranged as follows: for example, GFRP-7L-FPBT-DE-1S represents a specimen made of glass fiber-reinforced polymer (GFRP), consisting of seven layers (7L), tested under the four-point bending test (FPBT), kept in a dry environment (DE not exposed to seawater), and being the first specimen (1S). Similarly, GFRP-7L-FPBT-2M-1S indicates a specimen with the same structural features but immersed in seawater for two months (2M) before testing. For carbon fiber-reinforced specimens, CFRP-8L-FPBT-DE-1S denotes an eight-layer (8L) specimen tested under dry conditions (DE not exposed to seawater) and identified as the first specimen (1S) (Figure 10).

**Figure 11:**
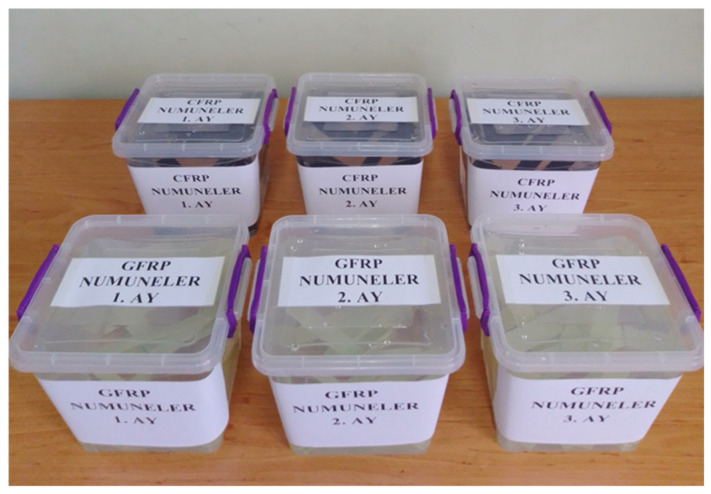
Soaking GFRP and CFRP samples in sea water for 1, 2 and 3 months for three-point bending.

**Figure 12:**
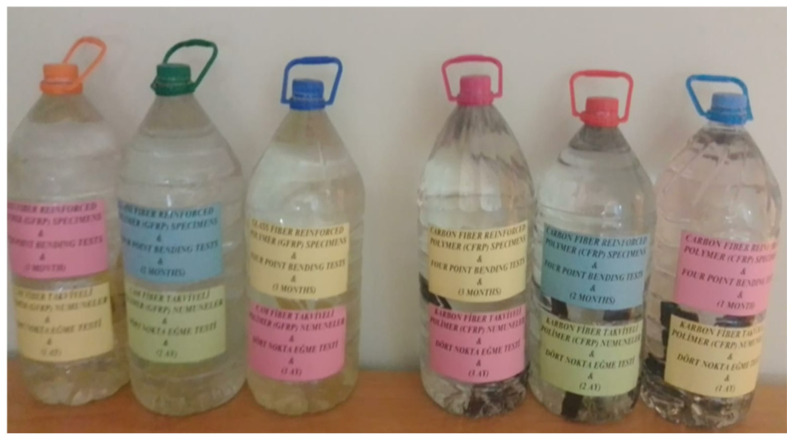
Soaking GFRP and CFRP samples in sea water for 1, 2 and 3 months for four-point bending tests.

Samples prepared using the single-lap method were first conditioned in a dry environment for three-point bending tests and then conditioned in seawater for periods of 1, 2, and 3 months (Figure 11). The same procedure was applied for the four-point bending tests. The seawater medium was prepared with a salinity range of 3.3–3.7% and a constant temperature of 22 °C, and all experiments were conducted under the same conditions (Figure 12).

### Comparative Analysis of Three- and Four-Point Bending Tests for GFRP and CFRP Composites in Marine Environment and Offshore Wind Turbine Blades

Flexural tests are commonly employed to evaluate the mechanical performance of materials and to determine their characteristic properties such as ductility, flexural strength, yield strength, elastic modulus, and fracture toughness. In the three-point bending test, the specimen is supported at both ends, and deformation is observed under a centrally applied load. This method allows the mechanical behavior of materials with different cross-sectional geometries to be analyzed under the assumption of a simple beam model, considering an ideal moment distribution and negligible shear stresses. In contrast, the four-point bending test involves supporting the specimen at both ends and applying two equal loads, thereby creating a constant moment region that enables a detailed examination of both elastic and plastic deformation behaviors. Accordingly, under dynamic bending type loading conditions, the mechanical reliability of the system is strongly influenced by the fiber matrix interfacial integrity of the composite structure [43].

**Figure 13:**
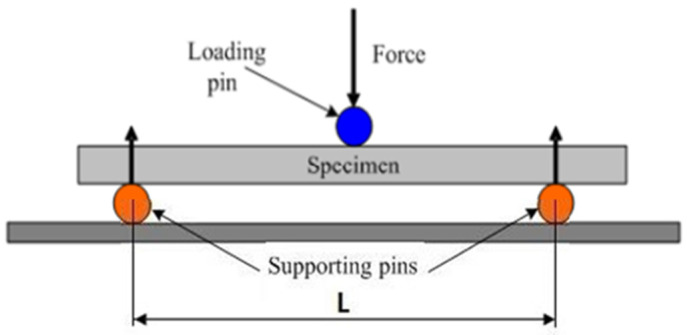
Schematic view of three-point bending test conditions.

In this study, the effects of adhesive type, joint geometry, and composite material type on mechanical performance were comprehensively investigated through both three-point and four-point bending tests. In the three-point bending test, the specimen was supported at both ends, and a load was applied at the center generated maximum stress; this condition caused the highest stress to occur in the outermost fibers at the midspan of the beam, thus identifying the region most susceptible to failure under bending (Figure 13). This test method is particularly useful for determining the damage mechanisms that occur in the adhesive interface and joint regions of single-lap GFRP and CFRP composite joints aged in marine environments, as well as for analyzing time-dependent variations in their mechanical properties.

**Figure 14:**
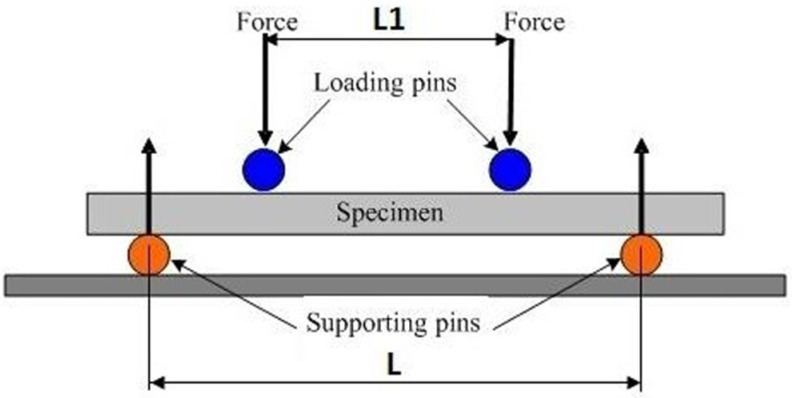
Schematic view of four-point bending test conditions.

On the other hand, the four-point bending test was applied to offshore wind turbine blade composites. The presence of a constant moment region enabled a detailed assessment of the specimen’s elastic and plastic behavior (Figure 14). During this test, the maximum stress and strain values occurring at the center of the adhesive-bonded joint specimens were calculated at each load level, allowing for a reliable evaluation of the mechanical performance of the composite joints. Both testing methods provide a systematic and comprehensive approach to analyzing the behavior of different composite materials and bonding parameters under marine environmental conditions.

The following formula is applied to calculate the amount of stress at a particular point on the load–deflection curve.

*Flexural Stress *(*σf*):

(1) $$\sigma_{f}=\frac{3PL}{2bd^{2}}$$

where:

$\sigma_{f}$: stress in the outer fibers at midpoint, MPa.
*P*: load at a given point on the load–deflection curve, N.
*L*: support span, mm.
*b*: width of beam tested, mm.
*d*: depth of beam tested, mm.

*Flexural Strain*, *εf*:

(2) $$\varepsilon_{f}=\frac{6Dd}{L^{2}}$$

where:

$\varepsilon_{f}$: strain in the outer surface, mm/mm.
*D*: maximum deflection of the center of the beam, mm.
*L*: support span, mm.
*d*: depth, mm.

During the test, the stress and strain values at the center of the adhesively bonded joint specimen were determined at each loading step in the four-point bending test, thus evaluating the material’s mechanical performance.

The following formula is used to calculate the maximum stress value in the area between the loading points in the four-point bending test.

(3) $$\sigma =\frac{3F(L-L1)}{2bh^{2}}$$

where:

*L*: span between supports, mm.
*L*1: distance between the applied forces, mm.
*b*: specimen width, mm.
*h*: specimen thickness, mm.
*F*: applied force, N.

The strain ε is calculated using the following formula:

(4) $$\varepsilon =\frac{6h\delta (L-L1)}{a(3L^{2}-4L1^{2})}$$

where:

ε: strain, mm/mm.
*h*: thickness of the specimen, mm.
*L*: support span, mm.
*L*1: half the loading span, mm.
δ: deflection at the middle of the span, mm.

Both the three-point and four-point bending tests were conducted in the Biomechanics Laboratory of Ege University, Department of Mechanical Engineering, using bending fixtures compatible with a 100 kN capacity Shimadzu AG-100 (Shimadzu, Kyoto, Japan) testing machine. The experiments were performed under a 5 kN load and a crosshead speed of 1 mm/min. All tests were carried out in accordance with the ASTM D790 standard [41] on smooth GFRP and CFRP single-lap joint specimens with an adhesive thickness of 0.2 mm. The specimens were tested under both dry and seawater-conditioned environments to investigate the effects of environmental exposure on mechanical performance.

During the three-point bending tests, stress–strain curves were obtained and analyzed based on the experimental data. In the four-point bending tests, parameters such as applied load, test speed, and specimen geometry were defined on the testing system, and measurements were automatically recorded through the test software.

**Figure 15:**
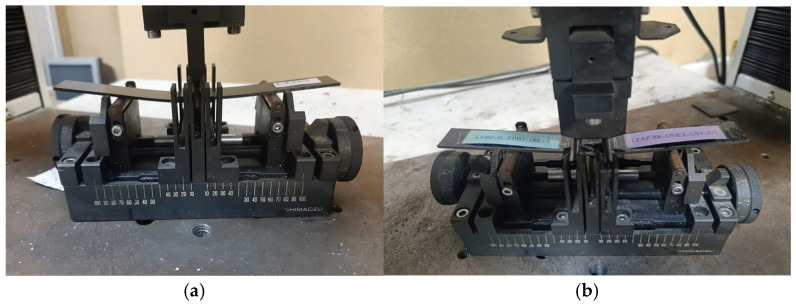
Placement of the sample in the three-point bending test (**a**); placement of the sample in the four-point bending test (**b**).

As a result, the influence of environmental factors on the flexural behavior of the adhesive interface was evaluated for all specimens, and variations in mechanical properties were comparatively analyzed based on the experimental findings. Figure 15 shows the placement of the specimens in the testing machine during both the three-point and four-point bending tests.

A total of 24 connection samples were used within the scope of the experiment, 12 of which were used for the three-point bending test and 12 for the four-point bending test.

## 3. Results

### 3.1. Three-Point Bending Test Results

#### GFRP and CFRP Samples

- ***Dry-Condition Reference Specimens: G-7-K and C-8-K***

**Figure 16:**
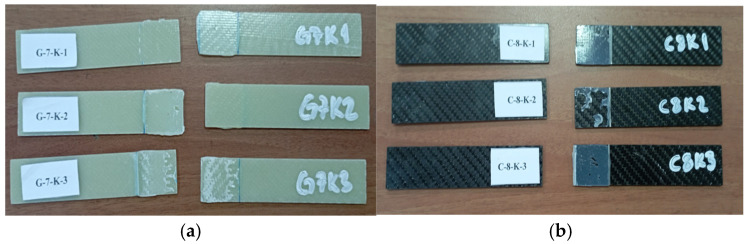
Views of the G-7-K sample after the three-point bending test (**a**) and the C-8-K sample after the test (**b**).

The damage patterns observed before and after the three-point bending tests applied to the G-7-K and C-8-K specimens, which were maintained under dry conditions, are presented in Figure 15. These specimens were used as reference samples for comparison with those exposed to seawater. The dry-condition specimens were stored at 22 °C and isolated from moisture and other environmental effects. Consequently, all observed deformations and damage can be attributed solely to the mechanical loading applied during the three-point bending test. As shown in Figure 16a, separations developed along the bond line in the G-7-K-1 and G-7-K-2 specimens. These separations are primarily associated with tensile stress concentrations at the specimen ends induced during bending. Similarly, Figure 16b indicates that failure initiated along the bond line in the C-8-K-2 and C-8-K-3 specimens. This behavior resulted from the localized maximum bending stress generated by the applied load. Despite the occurrence of fracture, the CFRP specimens largely preserved a smooth and bright surface morphology, indicating limited surface degradation under dry conditions.

- ***Reference Samples Preserved in Seawater for 1 Month: G-7-1A and C-8-1A***

**Figure 17:**
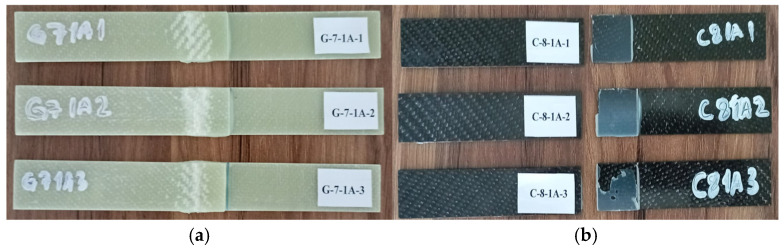
Views of the G-7-1A sample after the three-point bending test (**a**) and the C-8-1A sample after the test (**b**).

The damage patterns observed before and after the three-point bending test applied to the G-7-1A and C-8-1A specimens, which were immersed in seawater for one month, are presented in Figure 17.

As shown in Figure 17a, no pronounced fracture was observed along the bonding line of the G-7-1A specimens after one month of seawater exposure. Instead, whitish separation traces appeared, predominantly in the central regions of the specimens. These whitish zones are associated with localized micro-separations at the fiber–matrix interface of the GFRP material under bending loading. The relatively ductile behavior of the GFRP enabled partial dissipation of the applied load through localized deformation, thereby delaying complete fracture.

In contrast, as illustrated in Figure 17b, the C-8-1A-1 and C-8-1A-2 specimens exhibited sudden fractures propagating along the bonding line. The damage concentrated in the mid-span region indicates a predominantly brittle failure mechanism, where load transfer was abruptly interrupted. However, in the C-8-1A-3 specimen, partial adhesive residue remained attached to the specimen surface, suggesting that the interfacial bond was not entirely disrupted and that limited load transfer was maintained during failure.

- ***Reference Samples Stored in Seawater for 2 Months: G-7-2A and C-8-2A***

**Figure 18:**
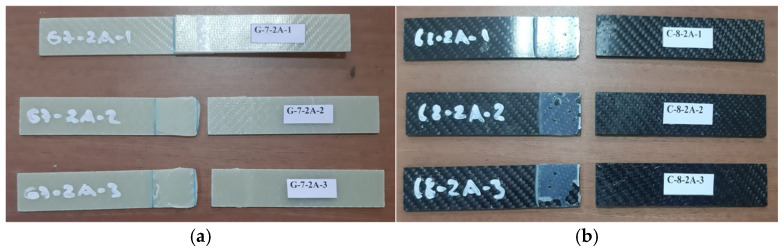
Views of G-7-2A sample (**a**) and C-8-1A sample (**b**) after three-point bending test.

Following the three-point bending tests, the damage modes observed in the G-7-2A and C-8-2A specimens exposed to seawater for two months were more pronounced and progressive compared to both the dry specimens and those exposed for one month. The fracture surface observations and SEM analyses presented in Figure 18 reveal that multiple damage mechanisms including interfacial weakening, cohesive failure within the adhesive, adhesive separation, matrix cracking, and localized delamination were simultaneously active in both composite systems.

In the GFRP-based G-7-2A specimens, the higher water absorption tendency of glass fibers promoted adhesive separation, microvoid formation, and the development of cohesive fracture zones along the joint line, which is consistent with the observed reduction in flexural strength. The delamination regions identified in specimens G-7-2A-2 and G-7-2A-3 are attributed to resin plasticization caused by prolonged moisture uptake and the consequent degradation of interfacial bond strength.

In the CFRP-based C-8-2A specimens, although carbon fibers exhibit a relatively low water absorption capacity, signs of fiber–matrix interfacial loosening, microcracking within the adhesive layer, and increased fiber pull-out were detected after two months of seawater exposure. The presence of adhesive residues and voided regions on the fracture surfaces of specimens C-8-2A-2 and C-8-2A-3 indicates a reduction in effective load transfer, which directly correlates with the strength degradation observed in the bending tests.

Overall, two months of seawater exposure led to more complex multimodal damage interactions and a more pronounced deterioration in mechanical performance compared to one month of exposure. These findings are consistently supported by both fracture surface examinations and SEM observations.

**Figure 19:**
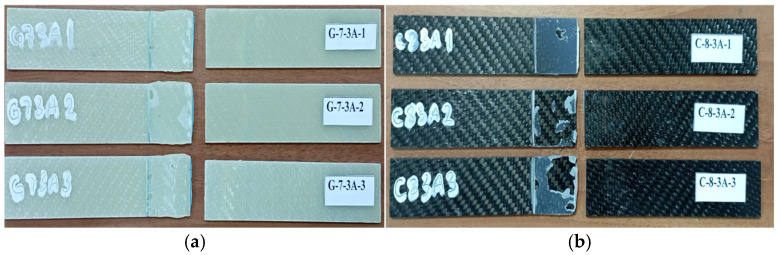
Views of G-7-3A sample (**a**) and C-8-3A sample (**b**) after three-point bending test.

As shown in Figure 19a, the G-7-3A specimen series exhibited multiple fracture modes, reflecting different levels of interfacial degradation after prolonged seawater exposure. In the G-7-3A-1 specimen, a fracture propagated directly along the bonding line, indicating a complete loss of adhesion in this region. The G-7-3A-2 specimen showed surface-level adhesive failure, which is attributed to insufficient bonding between the adhesive layer and the composite substrate. In contrast, the G-7-3A-3 specimen exhibited a mixed-mode fracture pattern, characterized by simultaneous interfacial failure and the presence of adhesive residues on the fracture surfaces. While damage in the first two specimens was mainly concentrated on the upper surfaces, the G-7-3A-3 specimen demonstrated fracture propagation across both adherend surfaces, suggesting a more distributed damage process.

- ***Reference Samples Stored in Seawater for 3 Months: G-7-3A and C-8-3A***

As illustrated in Figure 19b, the C-8-3A-1 specimen showed separation over a large portion of the adhesive layer; however, the overall structural integrity of the joint was largely preserved. The C-8-3A-2 specimen exhibited the most pronounced fracture and delamination features within the bonding region, indicating localized interfacial weakening. In the C-8-3A-3 specimen, partial adhesive residues remained on the fracture surface, suggesting that the adhesive substrate bond was not completely degraded and that partial load transfer was maintained until final failure.

**Figure 20:**
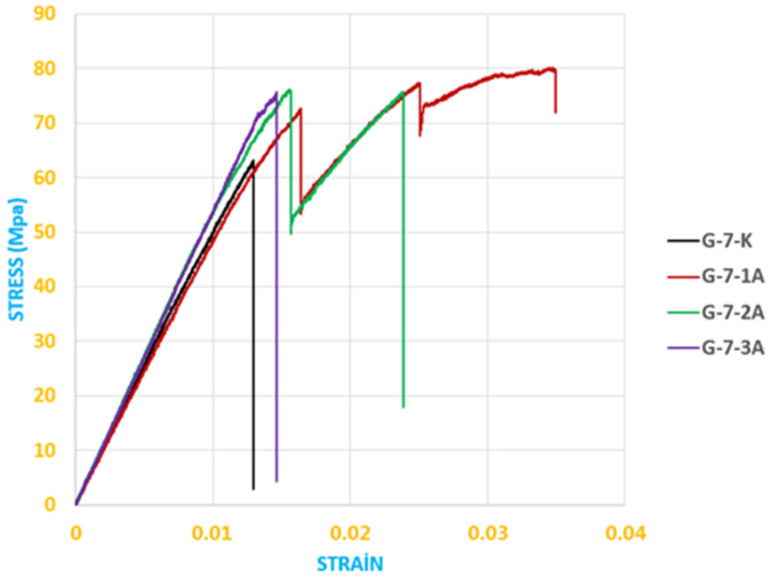
Stress–strain curves of GFRP specimens exposed to seawater for different durations and stored under dry conditions (three-point bending test).

The stress–strain responses presented in Figure 20 were obtained from three-point bending tests conducted on GFRP composite specimens stored under dry conditions and in seawater for exposure durations of 1, 2, and 3 months. These results provide a direct comparison of the mechanical response and degradation behavior as a function of environmental aging duration.

The data obtained from the bending stress–strain graph given in Figure 20 are shown in Table 2.

**Figure 21:**
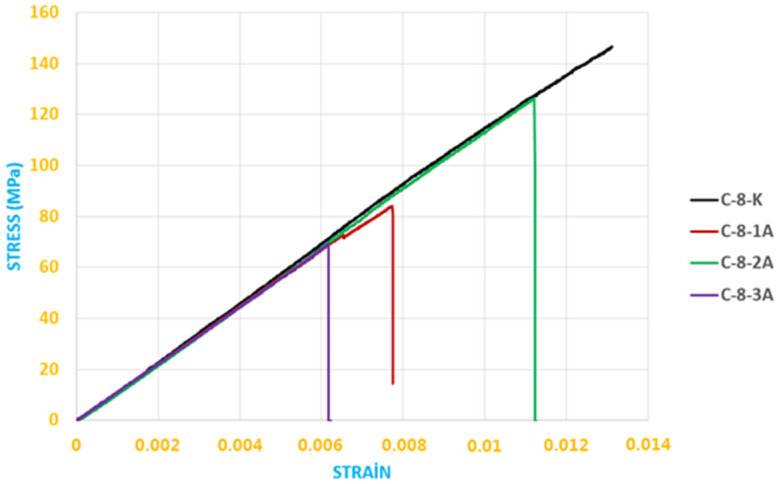
Stress–strain curves of CFRP specimens exposed to seawater for different durations and stored under dry conditions (three-point bending test).

The data obtained from the bending stress–strain graph given in Figure 21 are shown in Table 3.

The Elastic Modulus, also known as Young’s Modulus, is a fundamental mechanical property that defines how elastic or rigid a material behaves under external loads. This modulus is considered a quantitative indicator of a material’s stiffness. Materials with a high Young’s Modulus deform very little under applied force, indicating that they are stiffer and more rigid. Conversely, materials with a low elastic modulus tend to deform more under the same load and are therefore regarded as more flexible [43]. Here, the elastic modulus (E) values were calculated from the slope of the linear (elastic) regions of the stress–strain (*σ*–*ε*) curves. The elastic modulus values presented in Table 4 were obtained from the slope of the linear portion of the stress–strain data shown in Figure 20 and, similarly, from the slope of the linear portion of the data shown in Figure 21.

In Table 5 and Table 6, each value represents the mean of three independent samples. Standard deviation (SD) and coefficient of variation (CV) were calculated to evaluate the data distribution.

Table 5 and Table 6 present a comparative analysis of the mean Young’s (elastic) modulus values, standard deviation (SD), and coefficient of variation (CV) for GFRP and CFRP composite samples, respectively. The results show that CFRP samples exhibit significantly higher elastic modulus and more rigid mechanical behavior compared to GFRP samples. For both material types, a decrease in elastic modulus values was observed as the exposure time to seawater increased. However, this decrease was more pronounced in GFRP samples and more limited in CFRP samples. The results indicate that although CFRP samples exhibit higher standard deviation values compared to GFRP, the experiments generally demonstrate reproducible behavior. In particular, the lower CV values of CFRP samples reveal that this material exhibits more stable mechanical properties in challenging environmental conditions such as marine environments.

### 3.2. Four-Point Bending Test Results

#### GFRP and CFRP Samples

- ***Dry-Condition Reference Samples: GFRP-7L-FPBT-DE-1S and CFRP-8L-FPBT-DE-1S***

**Figure 22:**
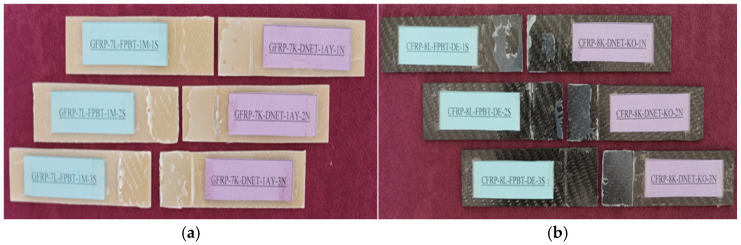
Views of the GFRP-7L-FPBT-DE-1S specimen (**a**) and CFRP-8L-FPBT-DE-1S specimen (**b**) after the four-point bending test.

In Figure 22a, damage in the GFRP-7L-FPBT-DE-1S specimens is observed to initiate along the adhesive bond line and progressively propagate toward the laminate surfaces. In regions where separation is concentrated, fine cracks advancing along the adhesive composite interface and partial interlaminar separations are identified. This behavior indicates that stress accumulation under bending loading becomes localized in specific regions, exceeding the strength limit of the adhesive bond line. As damage progresses, interlayer separation increases; however, the overall structural integrity of the specimen is preserved. This observation suggests that the failure process is not sudden but occurs in a gradual, interface-controlled manner.

In the CFRP-8L-FPBT-DE-1S specimens shown in Figure 22b, a similar fracture pattern was observed. Damage initiation predominantly occurred along the adhesive bond line and propagated toward the surface layers. In several specimens, remnants of the adhesive remained on the opposing fracture surfaces, indicating locally preserved adhesion. This observation suggests a mixed adhesive–cohesive failure mode. The localization of damage within a specific region indicates non-uniform load transfer along the bond line, with stress concentration in this area playing a dominant role in the fracture process.

- ***Reference Samples Preserved in Seawater for 1 Month: 1GFRP-7L-FPBT-1M and CFRP-8L-FPBT-1M***

**Figure 23:**
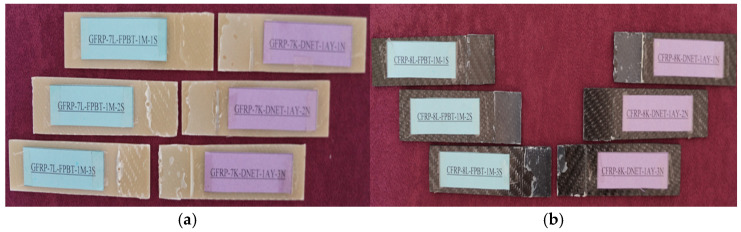
GFRP-7L-FPBT-1M specimen (**a**) and CFRP-8L-FPBT-1M specimen (**b**) after four-point bending tests.

In the GFRP-7L-FPBT-1M specimens shown in Figure 23a, a pronounced degradation at the adhesive interface was observed due to seawater exposure. Prolonged immersion in the saline environment facilitated the diffusion of water molecules and ions into the adhesive layer, leading to the weakening of chemical bonds and deterioration of adhesive integrity at the microscale. The accumulation of moisture and ions at the interface resulted in a reduction in the mechanical strength of the adhesive, promoting localized delamination and the formation of microcracks. Consequently, the adhesion strength along the bond line of the GFRP-7L-FPBT-1M specimens decreased, and fracture initiation predominantly occurred in these regions.

In contrast, the CFRP-8L-FPBT-1M specimens shown in Figure 23b exhibited a distinct damage mechanism. The inherently low water absorption capacity of carbon fiber-reinforced composites significantly restricted seawater ingress into the adhesive interface, allowing the adhesive composite bond to retain its stability over a longer exposure period. However, once failure occurred, it was characterized by a sudden and brittle fracture behavior. This response is attributed to the high elastic modulus and tensile strength of carbon fibers along the fiber direction. As a result, although the CFRP-8L-FPBT-1M specimens sustained higher fracture loads compared to the GFRP-7L-FPBT-1M specimens, failure occurred in a more abrupt manner.

- ***Reference Samples Stored in Seawater for 2 Months: GFRP-7L-FPBT-2M and CFRP-8L-FPBT-2M***

**Figure 24:**
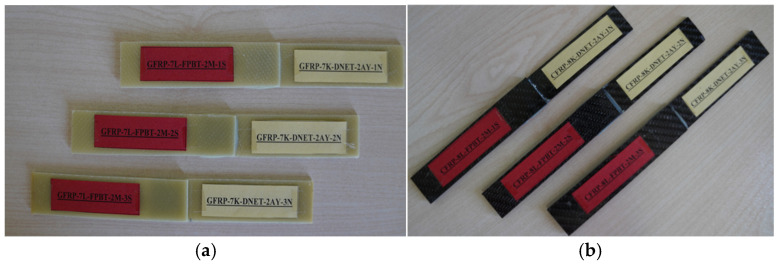
Views of the GFRP-7L-FPBT-2M specimen (**a**) and CFRP-8L-FPBT-2M specimen (**b**) after the four-point bending test.

In the GFRP-7L-FPBT-2M specimens shown in Figure 24a, damage was observed not only along the adhesive bond line but also in the form of adhesive residues remaining on both adherend surfaces. This observation indicates that fracture occurred both within the adhesive layer and at the adhesive substrate interface, corresponding to a mixed failure mode involving cohesive and adhesive mechanisms. During loading, the adhesive layer gradually lost its internal structural integrity, while the simultaneous reduction in interfacial adhesion forces led to separation between the bonded layers. The distribution of damage suggests that stresses along the bond line were non-uniform and that localized stress concentrations developed. Moreover, prolonged exposure to seawater induced both chemical degradation and physical weakening of the adhesive system, promoting the progression of mixed-mode fracture along the bond interface.

In the CFRP specimens shown in Figure 24b, a similar damage pattern was identified. The presence of adhesive residues on both bonding surfaces, together with fracture occurring within the adhesive layer, confirms the simultaneous development of cohesive and adhesive failure modes. However, a notable difference was observed in the CFRP-8L-FPBT-2M specimens, where the fiber-reinforced architecture enabled a more uniform load transfer along the bond line. The high elastic modulus and low water absorption of the carbon fibers restricted seawater ingress into the adhesive region, thereby contributing to the preservation of bonding integrity. Consequently, despite two months of seawater exposure, the bond line strength of the CFRP-8L-FPBT-2M specimens was largely maintained, and fracture occurred in a more controlled and stable manner.

- ***Reference Samples Stored in Seawater for 3 Months: GFRP-7L-FPBT-3M and CFRP-8L-FPBT-3M***

**Figure 25:**
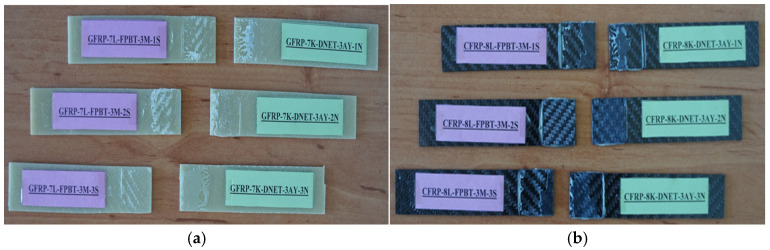
Views of GFRP-7L-FPBT-3M specimen (**a**) and CFRP-8L-FPBT-3M specimen (**b**) after four-point bending test.

In the GFRP-7L-FPBT-3M specimens shown in Figure 25a, which were immersed in seawater for three months, damage was observed to develop predominantly along the bond line. Fracturing was mainly concentrated in the central region of the joint, where adhesive residues partially remained on both bonded surfaces. This observation indicates that damage occurred primarily within the adhesive layer, accompanied by significant weakening of the adhesive–substrate interface. The diffusion of moisture and ions from seawater into the adhesive layer led to degradation and plasticization of the polymer matrix. As a result, microscopic voids and structural discontinuities formed within the adhesive and at the interface, reducing the mechanical integrity of the bond line. Consequently, the bond strength of the GFRP-7L-FPBT-3M specimens decreased markedly, and fracturing occurred at relatively lower load levels.

In the CFRP-8L-FPBT-3M specimens presented in Figure 25b, a similar damage mechanism was identified; however, the extent of degradation was considerably less pronounced. Following fracturing along the bond line, the presence of adhesive residues on both surfaces suggests the simultaneous occurrence of cohesive and adhesive failure modes. Owing to the high fiber strength and low water absorption of carbon fiber-reinforced composites, seawater penetration into the interfacial region was significantly restricted, allowing the bonding quality to be largely preserved. Therefore, despite prolonged seawater exposure, the CFRP-8L-FPBT-3M specimens maintained their structural integrity and exhibited higher bond strength compared to the GFRP-7L-FPBT-3M specimens.

**Figure 26:**
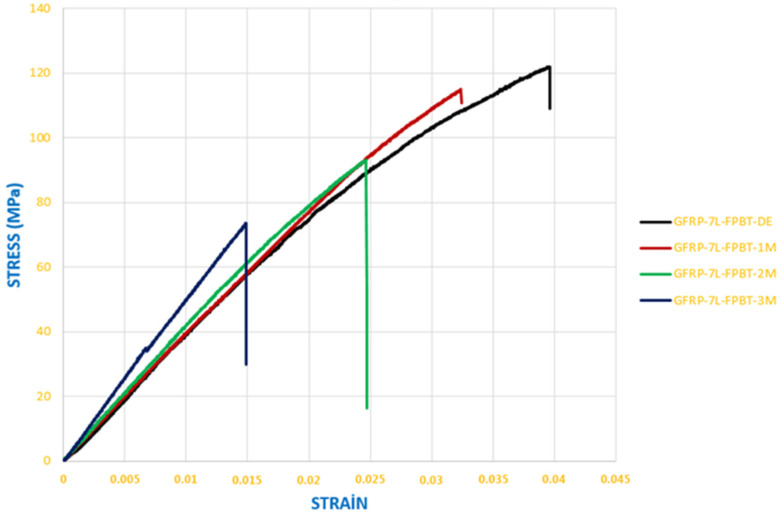
Stress–strain curves of GFRP specimens exposed to seawater for different durations and stored under dry conditions (four-point bending test).

Figure 26 presents the stress–strain results obtained from four-point bending tests conducted on GFRP composite specimens after being stored in dry conditions and in seawater for 1, 2, and 3 months.

The data obtained from the bending stress–strain graph given in Figure 26 are shown in Table 7.

**Figure 27:**
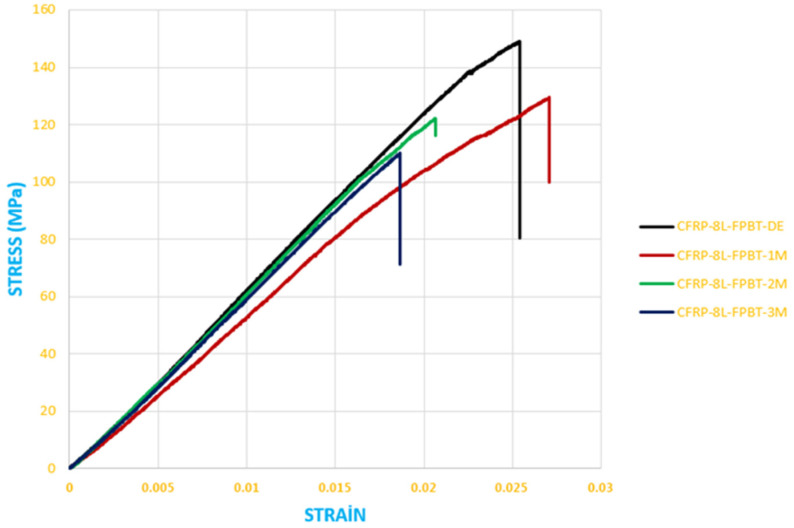
Stress–strain curves of CFRP specimens exposed to seawater for different durations and stored under dry conditions (four-point bending test).

The data obtained from the bending stress–strain graph given in Figure 27 are shown in Table 8.

**Figure 28:**
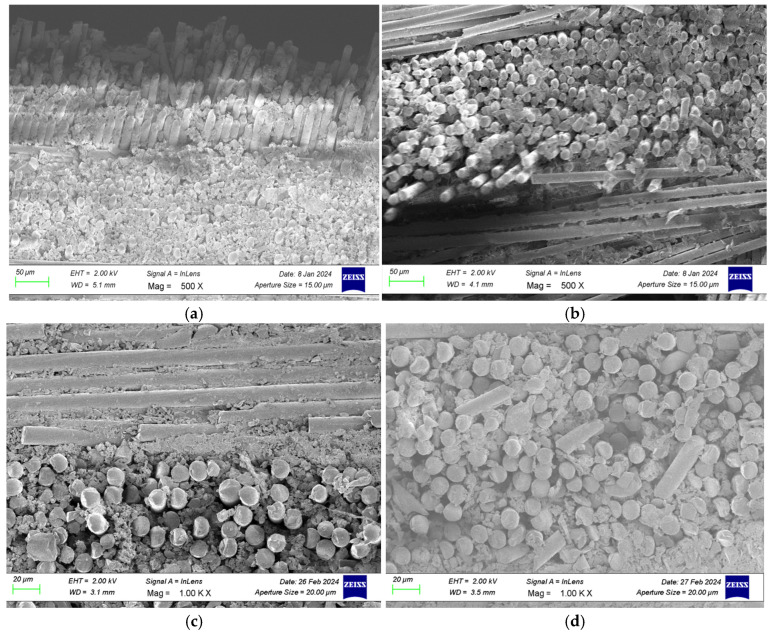
Reference (dry condition) GFRP specimens (**a**) and those exposed to seawater for 1 month (**b**), 2 months (**c**), and 3 months (**d**).

**Figure 29:**
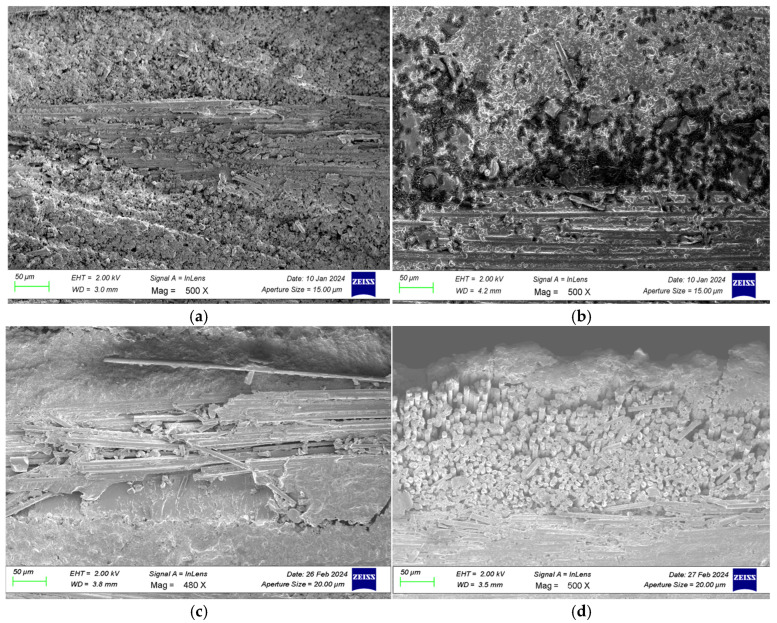
Reference (dry condition) CFRP specimens (**a**) and those exposed to seawater for 1 month (**b**), 2 months (**c**), and 3 months (**d**).

Here, the elasticity modulus (E) values were calculated from the slope of the linear (elastic) regions of the stress–strain (*σ*–*ε*) curves. The elasticity modulus values presented in Table 9 were obtained from the slope of the linear portion of the stress–strain data shown in Figure 28 and, similarly, from the linear portion of the data presented in Figure 29.

According to Table 9, a gradual decrease in the elasticity modulus was observed for both GFRP and CFRP specimens as the seawater exposure duration increased. This decrease was more pronounced in the GFRP specimens compared to the CFRP specimens. In particular, the reduction in the elasticity modulus of the G-7 series reached approximately 13% after three months of exposure, indicating the influence of water absorption in the matrix and the weakening of the fiber matrix interfacial bonding. In contrast, the reduction observed for the CFRP specimens remained within the range of 3–4%, demonstrating that carbon fibers exhibit a more resistant structure against moisture. These results reveal that CFRP maintains its mechanical stability under marine environmental conditions better than GFRP.

When Table 10 and Table 11 are examined together, it is seen that the elastic modulus values obtained within the scope of four-point bending tests show significant differences depending on material type and exposure time to seawater. In general, it was determined that the elastic modulus values of CFRP samples were higher than those of GFRP samples under all conditions. This reveals that CFRP composites exhibit a stiffer mechanical behavior thanks to the high elastic modulus and rigidity of carbon fibers.

Changes in elastic modulus values were observed as the exposure time to seawater increased for both material types. In GFRP samples, this change was more irregular and pronounced, and the deviations in elastic modulus values increased especially at two and three-month exposure times. This situation can be attributed to the higher water absorption tendency of glass fiber composites and the resulting weakening at the fiber–matrix interface. In CFRP samples, however, the elastic modulus values generally followed a more stable course, and the change remained limited despite the increasing exposure time. When standard deviation and coefficient of variation values are examined, it is observed that although the standard deviation is higher in CFRP samples in some cases compared to GFRP samples, the coefficient of variation remains at low levels, indicating that the obtained results are consistent in terms of average values and overall behavior. In particular, the low coefficient of variation obtained in CFRP samples after three months of seawater exposure reveals that this material exhibits more stable mechanical properties under challenging environmental conditions such as the marine environment.

In conclusion, the data obtained from four-point bending tests show that CFRP composites offer higher elastic modulus, better environmental resistance, and more stable mechanical behavior compared to GFRP composites. These findings reveal that CFRP materials are more suitable for long-term marine applications and applications requiring high structural rigidity.

The data presented in Table 12 include a comparative evaluation of the results obtained from three-point and four-point bending tests. Based on this comparison, Table 13 provides a performance analysis of the results from both testing methods, highlighting the differences in load-bearing capacity and deformation behavior of the specimens.

### 3.3. Damage Analysis of GFRP and CFRP Specimens in Three-Point and Four-Point Bending Tests

The three-point bending test is a widely used method for examining the flexural strength, fracture behavior, and interfacial interactions of composite materials. This test reveals the stress distribution in the tensile and compressive regions of the material, allowing for the evaluation of damage mechanisms, particularly at the fiber matrix interface.

However, the four-point bending test generates a wider pure bending region, enabling a more homogeneous examination of stress distribution along the material and providing more comprehensive information about interfacial strength and the overall integrity of the laminate structure.

As the duration of seawater exposure increases, interlaminar delamination, fibermatrix bond weakening, and damage development in the bonded regions become more pronounced in both tests. Such damage depends on multiple factors, including the microstructural characteristics of the material, the type of joint, and the chemical properties of the adhesive used. Therefore, microstructural analyses supported by three- and four-point bending tests are crucial for accurately assessing the performance changes of composite materials in marine environments.

SEM images of GFRP specimens after three-point bending, stored in dry conditions and in seawater for different durations (1, 2, and 3 months), are presented in Figure 24, while SEM images of CFRP specimens after three-point bending tests are shown in Figure 25. SEM images of GFRP specimens after four-point bending, under dry conditions and seawater exposure for 1, 2, and 3 months, are presented in Figure 26, and SEM images of CFRP specimens after four-point bending tests are shown in Figure 27.

#### 3.3.1. SEM Analysis of GFRP Specimens After Three-Point Bending Test

Within the scope of the three-point bending tests, the porosity values obtained from the SEM images of GFRP and CFRP specimens exposed to seawater for different durations are presented in Table 14 and Table 15, respectively. Porosity was determined through image analysis performed using ImageJ software (ImageJ.JS, web-based version; ImJoy; 2026). For each exposure duration, at least three different regions of interest (ROIs) were analyzed, and the results are reported as the mean ± standard deviation.

As shown in Figure 28a, for the GFRP specimens stored under dry conditions, i.e., without exposure to seawater, the three-point bending test revealed local fiber breakages and limited fiber pull-outs as the load increased. Such damage results from the fibers in the tensile and compressive regions being subjected to different stress levels under the applied bending load [44]. Since the specimens were in a dry environment, no chemical degradation, swelling, or structural weakening occurred at the fiber matrix interface, and therefore, the interfacial bonding strength was largely preserved. This indicates that strong load transfer between the fibers and the matrix was maintained.

As shown in Figure 28b, for specimens exposed to seawater for 1 month, the slow diffusion of water into the matrix caused weakening and microscale degradation at the fiber matrix interface. Ionic components in seawater chemically interacted with the epoxy matrix, reducing the bond strength and creating microvoids and detachments at the interface. This process led to fiber breakage under load and the formation of microcracks on the matrix surface. A slight increase in surface roughness was observed, making the material more susceptible to further water absorption in subsequent stages.

As shown in Figure 28c, for specimens exposed to seawater for 2 months, deeper penetration of water into the layers significantly weakened the fiber matrix interaction, and structural integrity began to deteriorate. At this stage, fiber fractures, matrix cracks, and delamination damage were clearly observed. Water progression at the fiber matrix interface facilitated crack propagation. Moreover, SEM images show that the surface had become more irregular compared to earlier stages, with an increase in microscale pits and fracture traces. This contributed to a reduction in mechanical strength.

As shown in Figure 28d, for specimens exposed to seawater for 3 months, extensive damage developed as moisture reached saturation within the material. Pronounced separations occurred at the fiber matrix interface, with voids and swelling forming around fiber bundles. Fiber breakages and crack propagation combined under load to create macro-scale delamination regions. Additionally, chemical degradation and water-induced swelling in the matrix significantly reduced interfacial bonding strength, leading to a considerable decline in the overall mechanical performance of the material. Although water absorption slowed in later stages, the structural degradation process did not stop; instead, the existing damage expanded and merged, further compromising the integrity of the material.

#### 3.3.2. SEM Examination of CFRP Specimens After Three-Point Bending Test

As shown in Figure 29a, for CFRP specimens stored under dry conditions (without exposure to seawater), the fibers were observed to be regularly aligned and exhibited strong bonding with the epoxy matrix. The matrix phase formed a homogeneous and continuous structure, with no voids, bubbles, or interfacial separations detected between the fiber bundles. However, as a result of the applied three-point bending load, local fiber breakages and fractures occurred in regions where load transfer was most concentrated. These fractures developed due to the high stresses experienced by the fibers in the tensile regions.

As shown in Figure 29b, for specimens exposed to seawater for 1 month, initial signs of microstructural changes were observed, indicating the onset of early damage. Diffusion of seawater into the matrix caused microscale irregularities on the matrix surface, slight swelling, and increased surface roughness. Softening of the epoxy matrix occurred with water absorption, and microscopic separations at the fiber matrix interface were detected. Small voids around the fibers partially weakened load transfer and resulted in limited fiber breakages. At this stage, the observed damage remained largely superficial due to the restricted penetration of water into the matrix.

As shown in Figure 29c, for specimens exposed to seawater for 2 months, the damage types became more pronounced, and various microstructural deformations appeared in the material. Seawater penetration into the matrix weakened the chemical bonding of the epoxy chains, leading to the development of microcracks and localized deterioration. Wider separation zones were detected at the fiber matrix interfaces, and in some areas, fiber bundles began to detach from the surface. Additionally, capillary-driven water movement along the interface resulted in the formation of micropores and bubbles.

As shown in Figure 29d, for specimens exposed to seawater for 3 months, microstructural deterioration reached an advanced stage. Pronounced cracks, localized delamination, and surface peeling occurred in the matrix phase. The size of the separations at the fiber matrix interface increased, with some carbon fibers protruding from the matrix and exposed at the surface. Prolonged seawater exposure led to the development of micropores and capillary voids within the matrix, facilitating water penetration into the interior and accelerating the damage process.

#### 3.3.3. SEM Analysis of GFRP Specimens After Four-Point Bending Test

Within the scope of the four-point bending tests, the porosity values obtained from the SEM images of GFRP and CFRP samples exposed to seawater for different durations are presented in Table 16 and Table 17, respectively. Porosity was determined through image analysis performed using ImageJ software. For each exposure duration, at least three different regions of interest (ROIs) were analyzed, and the results are reported as the mean ± standard deviation.

**Figure 30:**
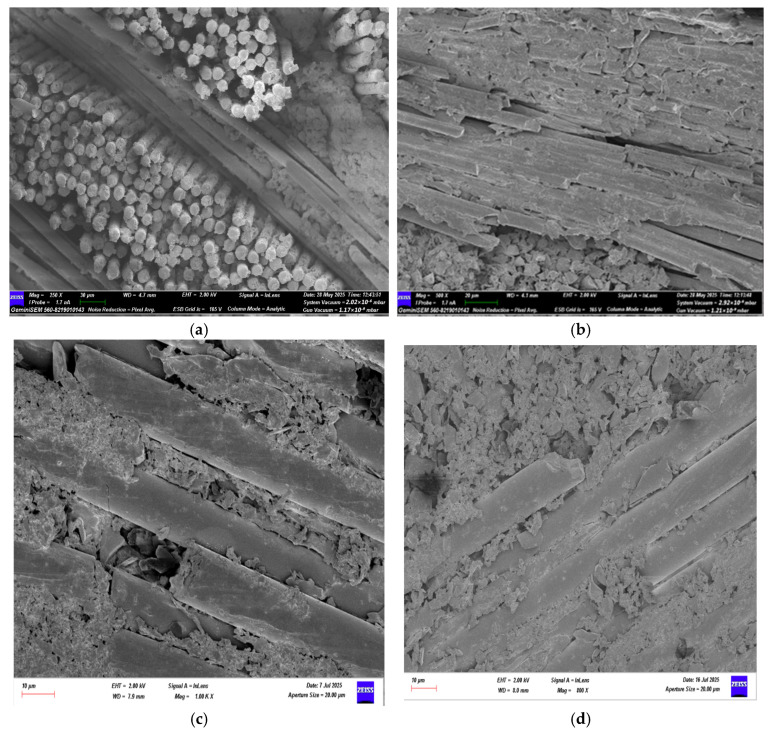
Reference (dry condition) GFRP specimens (**a**) and those exposed to seawater for 1 month (**b**), 2 months (**c**), and 3 months (**d**).

As shown in Figure 30a, the SEM images of the GFRP control specimen stored under dry conditions show strong bonding between the glass fibers and the polymer matrix. The fibers are evenly distributed, with minimal voids between them, and the fiber–matrix interface maintains its continuity. The absence of cracks or fracture traces in the fibers indicates that the specimen’s flexural strength remains high.

As shown in Figure 30b, for specimens exposed to seawater for 1 month, weakening of the bond between fibers and matrix is noticeable. Interfacial separations and fiber pull-outs occur, while microcracks and localized plastic deformations are observed on the matrix surface. Penetration of water molecules from seawater into the matrix causes swelling and stress accumulation in the material, reducing the interfacial bonding strength. Although the mechanical properties have decreased compared to the dry condition, the structural integrity is partially preserved.

As shown in Figure 30c, for GFRP specimens exposed to seawater for 2 months, microstructural deterioration becomes more pronounced. Fiber breakages and shrinkages, along with numerous microcracks, voids, and fragmented areas within the matrix, are observed. Fiber matrix interaction is largely lost, and interfacial bond failure limits load transfer. This results in reduced flexural strength and the emergence of brittle behavior in the specimen.

As shown in Figure 30d, for specimens exposed to seawater for 3 months, the microstructural integrity is almost completely lost. The fibers have detached from the matrix, becoming free, and severe chemical degradation has occurred in the polymer matrix. Microscale cracks have coalesced into macroscopic fractures over time, leading to sudden and brittle failure. The mechanical strength of the matrix has been significantly reduced due to water exposure.

#### 3.3.4. SEM Analysis of CFRP Specimens After Four-Point Bending Test

**Figure 31:**
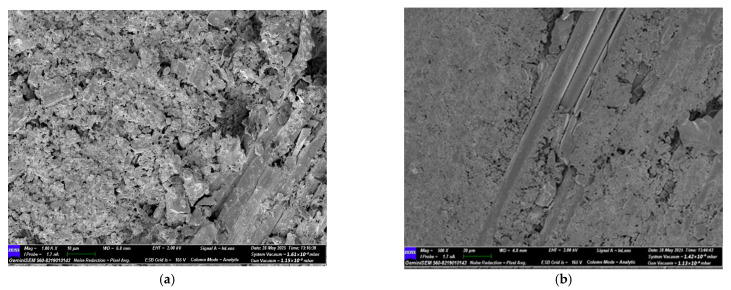

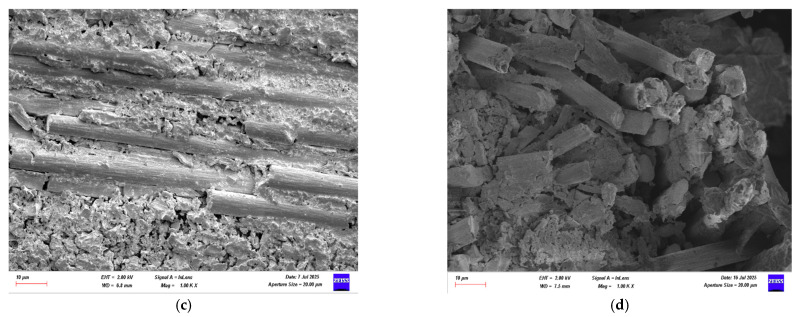
Reference (dry condition) CFRP specimens (**a**) and those exposed to seawater for 1 month (**b**), 2 months (**c**), and 3 months (**d**).

As shown in Figure 31a, the SEM images of the CFRP control specimen stored under dry conditions show strong bonding between the fibers and the matrix. The fibers are properly embedded in the matrix, exhibiting a regular structure and high interfacial integrity at the fracture surface. Some fiber pull-out is observed, but overall the fracture occurs in a balanced manner. This indicates that energy during mechanical loading is dissipated in a ductile way, and the fracture occurs in a controlled rather than sudden manner.

As shown in Figure 31b, for CFRP specimens exposed to seawater for 1 month, initial microstructural deterioration is observed. Local separations appear in the interfacial regions, and microcracks and deformation traces become noticeable on the matrix surface. These changes indicate that the fiber matrix bonds are beginning to weaken, and the mechanical properties of the specimen show a decreasing trend.

As shown in Figure 31c, after two months of seawater exposure, structural deterioration becomes more advanced. Fibers detach from the matrix, and swelling, cracking, and voids become more pronounced in the resin phase. The fiber fractures appear short and irregular, indicating that the fracture surface is gradually gaining a more brittle character.

As shown in Figure 31d, after three months of seawater exposure, the microstructural integrity is significantly weakened. Bonds at the fiber matrix interface are largely loosened, and delamination and void formation in the resin layer are increased. These developments result in a considerable reduction in the mechanical strength of the composite structure. Table 18 presents a comparison of the SEM results for GFRP and CFRP specimens after three-point and four-point bending tests.

SEM analyses revealed that, in both GFRP and CFRP specimens, microstructural deterioration became increasingly pronounced as the duration of seawater exposure increased. Under dry conditions, high interfacial integrity and strong fiber matrix interaction were observed. However, starting from the first month of exposure, interfacial degradation, microcracks, and void formation occurred due to water diffusion into the interface. This process led to increased chemical degradation and stress accumulation in the matrix, weakening the interfacial bonding.

During the two- and three-month aging periods, both glass fiber (GFRP) and carbon fiber (CFRP) specimens exhibited reduced fiber adhesion to the matrix, expansion of delamination regions, and a shift in fracture behavior from ductile to brittle. In GFRP specimens, deterioration primarily manifested as matrix deformation and interfacial debonding, whereas in CFRP specimens, microvoid formation and surface separations became more pronounced.

In four-point bending tests, the wider load transfer regions allowed structural damage to distribute more evenly, while in three point bending tests, stress concentration at a single point caused damage to occur more locally and suddenly. Consequently, prolonged seawater exposure weakened the fiber matrix bond strength, disrupted microstructural integrity, and led to a significant reduction in flexural strength in both GFRP and CFRP composites.

## 4. Discussion and Conclusions

The strength of adhesively bonded single-lap GFRP and CFRP joints used in marine environments, particularly in offshore wind turbine blades, is critically dependent on long-term exposure to seawater. Existing studies have generally been conducted under single-material conditions and limited environmental scenarios, leaving the effects of different composite types and seawater exposure largely unexplored.

In this study, GFRP and CFRP specimens were stored both under dry conditions and in natural seawater collected from the Aegean Sea (22 °C, 3.3–3.7% salinity) for 1, 2, and 3 months. Their mechanical behavior and damage characteristics were comparatively evaluated through three-point and four-point bending tests.

The results from the three-point bending tests showed that the Young’s modulus of GFRP decreased by approximately 13% after 3 months of seawater exposure, whereas the reduction observed for CFRP was limited to only 3.7%. In GFRP, micro-separations, partial fiber pull-outs, and matrix deformation were observed, while CFRP exhibited minimal damage, maintaining better structural strength. SEM analyses revealed that, in both materials, damage initiated in the matrix phase before propagating to the fiber phase, with CFRP maintaining a more uniform fiber matrix interfacial bond compared to GFRP.

A similar trend was observed in the four-point bending tests. GFRP’s Young’s modulus decreased by 9.5%, whereas the reduction observed for CFRP remained limited to 3.5%. In GFRP, cracks and layer delamination progressed gradually, whereas CFRP demonstrated more homogeneous load transfer and controlled fracturing. Seawater exposure in GFRP led to interfacial weakening and microvoid formation, whereas the adhesive–layer bond in CFRP remained largely intact, resulting in fracturing at higher load levels. The use of F-RES 21 epoxy resin and F-Hard 22 hardener contributed to preserving structural integrity in both material groups.

Overall, both three-point and four-point bending tests demonstrated that CFRP joints are more resistant to seawater exposure compared to GFRP joints, offering higher mechanical strength and structural stability. While stress concentration at a single point in three-point bending led to local and sudden damage, the broader load transfer in four-point bending allowed for more uniform distribution of damage.

## Figures and Tables

**Table 1:** Coding System for GFRP and CFRP Specimens for Three- and Four-Point Bending Tests.

Code	Test Type	Description
G-7-K-1	Three-Point Bending	Glass fiber (G), 7 layers, tested in dry condition(K), specimen 1
G-7-1A-1	Three-Point Bending	Glass fiber(G), 7 layers, conditioned in seawater for 1 month (1A), specimen 1
C-8-K-1	Three-Point Bending	Carbon fiber (C), 8 layers, tested in dry condition(K), specimen 1
GFRP-7L-FPBT-DE-1S	Four-Point Bending	Glass fiber (GFRP), 7 layers (7L), four-point bending test (FPBT), dry condition (DE), specimen 1(1S)
GFRP-7L-FPBT-2M-1S	Four-Point Bending	Glass fiber (GFRP), 7 layers (7L), four-point bending test (FPBT), 2 months in seawater (2M), specimen 1 (1S)
CFRP-8L-FPBT-DE-1S	Four-Point Bending	Carbon fiber (CFRP), 8 layers (8L), four-point bending test (FPBT), dry condition (DE), specimen 1(1S)

**Table 2:** Bending Stress and Strain Values of GFRP Specimens.

Specimen Code	Exposure Time	Flexural Stress (MPa)	Strain (*ε*)	Description/Status
G-7-K	Dry (Reference)	63.0726	0.0129	Maximum stress value
G-7-1A	1 month	72.2704	0.0163	Initial stress
		54.4886	0.0113	Minimum stress
		80.0818	0.0347	Maximum stress
G-7-2A	2 months	75.8136	0.0156	Initial maximum stress
		50.8163	0.0094	Stress reduction
		75.6766	0.0154	Stress increase
G-7-3A	3 months	75.6723	0.0146	Initial maximum stress

Note: Three independent samples were tested for each exposure time. The values in Table 2 are the average of three samples.

**Table 3:** Bending Stress and Strain Values of CFRP Specimens.

Specimen Code	Exposure Time	Flexural Stress (MPa)	Strain (*ε*)	Description/Status
C-8-K	Dry (Reference)	146.5976	0.0131	Maximum bending stress
C-8-1A	1 month	72.4395	0.0065	Initial stress
		83.6555	0.0077	Maximum stress
C-8-2A	2 months	126.2889	0.0112	Initial and maximum stress
C-8-3A	3 months	69.1586	0.0061	Bending stress

Note: Three independent samples were tested for each exposure time. The values in Table 3 are the average of three samples.

**Table 4:** Young’s Modulus (E) of GFRP and CFRP specimens.

Sample Code	Material Type	Elastic Modulus (E) (GPa)	Change Compared to Reference (%)
G-7-K	GFRP	5.39	Dry environment (reference)
G-7-1A	GFRP	5.07	% 5.94
G-7-2A	GFRP	4.91	% 8.90
G-7-3A	GFRP	4.69	% 12.98
C-8-K	CFRP	11.50	Dry environment (reference)
C-8-1A	CFRP	11.36	% 1.28
C-8-2A	CFRP	11.11	% 3.39
C-8-3A	CFRP	11.07	% 3.74

Note: Three independent samples were tested for each exposure time. The values in Table 4 are the average of three samples.

**Table 5:** Mean, SD, and CV values of strength and elastic modulus obtained from 3-point bending tests on GFRP samples.

Sample Code	Material Type	Elastic Modulus (E) (GPa)	SD (GPa)	CV (%)
G-7-K	GFRP	5.39	0.61	11.40
G-7-1A	GFRP	5.07	0.42	8.28
G-7-2A	GFRP	4.91	0.44	8.90
G-7-3A	GFRP	4.69	0.61	12.98

**Table 6:** Mean, SD, and CV values of strength and elastic modulus obtained from 3-point bending tests on CFRP samples.

Sample Code	Material Type	Elastic Modulus (E) (GPa)	SD (GPa)	CV (%)
C-8-K	CFRP	11.50	0.79	7.03
C-8-1A	CFRP	11.36	0.63	5.97
C-8-2A	CFRP	11.11	0.17	1.53
C-8-3A	CFRP	11.07	0.46	3.94

**Table 7:** Bending Stress and Strain Values of GFRP Specimens.

Specimen Code	Exposure Time	Flexural Stress (MPa)	Strain (*ε*)	Description/Status
GFRP-7L-FPBT-DE	Dry (Reference)	121.6930	0.0395	Maximum bending stress
GFRP-7L-FPBT-1M	1 month	114.9519	0.0323	Initial stress
GFRP-7L-FPBT-2M	2 months	92.6155	0.0244	Highest stress
GFRP-7L-FPBT-3M	3 months	72.7945	0.0146	Lowest stress

Note: Three independent samples were tested for each exposure time. The values in Table 7 are the average of three samples.

**Table 8:** Bending Stress and Strain Values of CFRP Samples.

Specimen Code	Exposure Time	Flexural Stress (MPa)	Strain (*ε*)	Description/Status
CFRP-8L-FPBT-DE	Dry (Reference)	148.5722	0.0254	Maximum bending stress
CFRP-8L-FPBT-1M	1 month	12.2385	0.0270	Slight increase in flexibility at the beginning (matrix softening)
CFRP-8L-FPBT-2M	2 months	121.9446	0.0206	Tendency of stress reduction
CFRP-8L-FPBT-3M	3 months	109.5578	0.0185	Noticeable mechanical degradation

Note: Three independent samples were tested for each exposure time. The values in Table 8 are the average of three samples.

**Table 9:** Young’s Modulus (E) of GFRP and CFRP specimens.

Sample Code	Material Type	Elastic Modulus (E) (GPa)	Change Compared to Reference (%)
GFRP-7L-FPBT-DE	GFRP	3.840	Dry environment (reference)
GFRP-7L-FPBT-1M	GFRP	3.856	% 3.15
GFRP-7L-FPBT-2M	GFRP	3.843	% 6.42
GFRP-7L-FPBT-3M	GFRP	5.213	% 9.45
CFRP-8L-FPBT-DE	CFRP	6.270	Dry environment (reference)
CFRP-8L-FPBT-1M	CFRP	5.380	% 1.29
CFRP-8L-FPBT-2M	CFRP	6.169	% 2.62
CFRP-8L-FPBT-3M	CFRP	6.036	% 3.48

Note: Three independent samples were tested for each exposure time. The values in Table 9 are the average of three samples.

**Table 10:** Mean, SD, and CV values of strength and elastic modulus obtained from 4-point bending tests on GFRP samples.

Sample Code	Material Type	Elastic Modulus (E) (GPa)	SD (GPa)	CV (%)
GFRP-7L-FPBT-DE	GFRP	3.840	0.21	2.66
GFRP-7L-FPBT-1M	GFRP	3.856	0.19	2.77
GFRP-7L-FPBT-2M	GFRP	3.843	0.74	12.40
GFRP-7L-FPBT-3M	GFRP	5.213	0.33	5.64

**Table 11:** Mean, SD, and CV values of strength and elastic modulus obtained from 4-point bending tests on CFRP samples.

Sample Code	Material Type	Elastic Modulus (E) (GPa)	SD (GPa)	CV (%)
CFRP-8L-FPBT-DE	CFRP	6.270	0.58	5.77
CFRP-8L-FPBT-1M	CFRP	5.380	0.83	8.75
CFRP-8L-FPBT-2M	CFRP	6.169	0.747	7.10
CFRP-8L-FPBT-3M	CFRP	6.036	0.331	0.03

**Table 12:** Comparison of Results of Three-Point Bending and Four-Point Bending Tests.

Condition	Three-Point Bending Results	Four-Point Bending Results
Dry Environment (Reference)	The G-7-K and C-8-K specimens were isolated from environmental effects. Damage occurred solely due to mechanical bending. In G-7-K, separations along the bond line caused by tensile stresses were observed. In the C-8-K specimens, surface gloss and integrity were preserved.	In the GFRP-7L-FPBT-DE-1S and CFRP-8L-FPBT-DE-1S specimens, damage initiated at the bond line and progressed towards the surfaces. Gradual (not sudden) fracture was observed. Stress concentration was evident in the CFRP-8L-FPBT-DE-1S specimens.
Samples Soaked in Seawater for 1 Month	G-7-1A: No significant rupture at the bond line, but micro-separations (whitening) occurred at the fiber–matrix interface. G-7-1A demonstrated a certain degree of ductility. C-8-1A: Sudden and brittle ruptures were observed at the bond line, with adhesion partially preserved in some samples.	GFRP-7L-FPBT-1M: Weakening, microcracks, and delamination occurred at the interface due to the ionization effect of seawater. CFRP-8L-FPBT-1M: Due to low water absorption, the bond line maintained its stability, but it exhibited brittle behavior at the time of fracture.
Samples Soaked in Seawater for 2 Months	GFRP (G-7-2A): Significant fractures and bond weaknesses were observed along the bond line. CFRP (C-8-2A): Local cracks, adhesive separation, and partial voids were observed at the bond line. The structural integrity of the CFRP specimens was better preserved than that of the GFRP specimens.	GFRP-7L-FPBT-2M: Adhesive residue was observed at the bond line and on the surfaces. Mixed failure (adhesive + cohesive) occurred. CFRP-8L-FPBT-2M: Similar mixed failure was observed. Fiber reinforcement provided more balanced load transfer.
Samples Soaked in Seawater for 3 Months	GFRP (G-7-3A): Damage progressed directly along the bond line, decreasing bond line strength. The adhesive layer became plastic, creating microscopic voids. CFRP (C-8-3A): Similar damage but more limited; cohesive and adhesive failure were observed simultaneously.	GFRP-7L-FPBT-3M: Damage was concentrated at the bond line, and the adhesive was observed to have partially adhered to both surfaces. Strength decreased due to seawater weakening the polymer structure. CFRP-8L-FPBT-3M: Similar fracture type but less severe damage. Bond quality was largely maintained.

**Table 13:** Performance Analysis of Three-Point and Four-Point Bending Test Results.

Observation	Three-Point Bending	Four-Point Bending
Damage Initiation	Typically initiates along the bond line, especially at the edge regions.	Initiates at the bond line and propagates toward the surfaces.
Type of Damage	Over time, adhesive and cohesive failures are observed simultaneously.	Mixed-mode failure (adhesive + cohesive) is dominant.
Effect of Seawater	Rapid degradation is seen in GFRP specimens, while the effect is limited in CFRP specimens.	In GFRP specimens, chemical and physical degradation occurs, whereas in CFRP specimens, low water absorption helps preserve strength.
Fracture Behavior	GFRP specimens exhibit ductile–mixed behavior; CFRP specimens show brittle behavior.	GFRP: delamination observed; CFRP: sudden but controlled fracture observed.
Preservation of Structural Strength	CFRP > GFRP	CFRP > GFRP
Result of Long-Term Exposure	Adhesive strength decreases significantly in GFRP specimens.	CFRP specimens maintain structural integrity for a longer period.

**Table 14:** Porosity values of GFRP specimens calculated from SEM images for different exposure durations (mean ± standard deviation, n = 3) (3-point bending representative porosity values).

Exposure	Porosity (%)
1 month in seawater	1.05 ± 0.12
2 months in seawater	1.32 ± 0.15
3 months in seawater	1.19 ± 0.10

**Table 15:** Porosity values of CFRP specimens calculated from SEM images for different exposure durations (mean ± standard deviation, n = 3) (3-point bending representative porosity values).

Exposure	Porosity (%)
1 month in seawater	0.36 ± 0.07
2 months in seawater	0.53 ± 0.10
3 months in seawater	0.19 ± 0.09

**Table 16:** Porosity values of GFRP specimens calculated from SEM images for different exposure durations (mean ± standard deviation, n = 3) (4-point bending representative porosity values).

Exposure	Porosity (%)
1 month in seawater	1.08 ± 0.13
2 months in seawater	1.35 ± 0.16
3 months in seawater	1.22 ± 0.12

**Table 17:** Porosity values of CFRP specimens calculated from SEM images for different exposure durations (mean ± standard deviation, n = 3) (4-point bending representative porosity values).

Exposure	Porosity (%)
1 month in seawater	0.38 ± 0.09
2 months in seawater	0.54 ± 0.10
3 months in seawater	0.20 ± 0.08

**Table 18:** Comparison of SEM Results from Three-Point and Four-Point Bending Tests.

Condition	GFRP (Three-Point Bending)	GFRP (Four-Point Bending)	CFRP (Three-Point Bending)	CFRP (Four-Point Bending)
Dry Environment (Reference)	Local fiber fractures and limited pull-outs were observed. No chemical degradation or swelling occurred. The fiber matrix bond is strong, and load transfer is effective.	The fiber matrix interface is strong. Fibers are evenly distributed with a low void content. The fracture exhibited ductile behavior.	The fibers are observed to be regularly aligned, and the matrix is homogeneous. Fiber breakages at the interface are limited.	The fiber matrix interface exhibits high integrity. Fibers are firmly bonded to the matrix, and fracture occurs in a controlled manner.
Samples Soaked in Seawater for 1 Month	Diffusion of water into the matrix weakened the interface, leading to the formation of microcracks and voids. Surface roughness has increased.	Degradation has begun in the fiber matrix bonds. Microcracks and plastic deformations are observed.	Irregularities and swelling are observed on the matrix surface. Microscopic separations have begun at the fiber matrix interface.	Local separations and microcracks have formed at the interface. Matrix deformations have become pronounced, and bond strength has decreased.
Samples Soaked in Seawater for 2 Months	Fiber matrix interaction has weakened, with increased delamination and microcracks. The surface has become irregular.	Microstructural deterioration has become pronounced. Fiber fractures and void formation are observed.	The chemical bonds have weakened, leading to the formation of microcracks and surface separations. Fiber bundles have partially detached.	Fibers have detached from the matrix, and swelling and voids in the resin phase have intensified. The fracture behavior has become more brittle.
Samples Soaked in Seawater for 3 Months	Pronounced separations and voids have formed at the fiber matrix interface. Fiber fractures have coalesced, creating delamination regions.	The microstructural integrity is largely lost. Fibers have become free, and the matrix has undergone chemical degradation.	Matrix cracks and surface peeling have increased. Some fibers have lost their adhesion to the matrix.	Fiber matrix bonds have separated, and delamination and voids in the resin structure have become pronounced. Mechanical strength has decreased significantly.

## Data Availability

The original contributions presented in the study are included in the article, further inquiries can be directed to the corresponding author.
